# Controlled and Safe Hydrogen Generation from Waste Aluminum and Water, a New Approach to Hydrogen Generation

**DOI:** 10.3390/ma17235885

**Published:** 2024-11-30

**Authors:** Xavier Salueña-Berna, Marc Marín-Genescà, Ramon Mujal Rosas, Manuel-Jose Lis Arias

**Affiliations:** 1 Mechanical Engineering Department, ESEIAAT-UPC, Colom 11, 08222 Terrassa, Spain; xavier.saluena@upc.edu; 2 Mechanical Engineering Department, ETSEQ-URV, Països Catalans 26, 43007 Tarragona, Spain; 3 Electric Engineering Department, ETSEIAAT-UPC, Colom 11, 08222 Terrassa, Spain; ramon.mujal@upc.edu; 4 Chemical Engineering Department, ESEIAAT-UPC, Colom 11, 08222 Terrassa, Spain; manuel-jose.lis@upc.edu

**Keywords:** aluminum breakage scrap, sustained hydrogen, hydrogen generation, aluminum–water apparatus, portable hydrogen generation, hydrogen reactor, aluminum–water reaction, Al + NaOH test tubes

## Abstract

A new method is proposed to generate hydrogen in situ at low pressure from powder-pressed recycled aluminum turnings activated with small amounts of NaOH and drops of water. The contribution of this system is that the user can obtain small flows of high-purity hydrogen (>99%) to charge their portable electronic devices in remote places, in a simple, controlled, and safe way, since only water is used. Test tubes that contain tiny amounts of NaOH on their surface can be transported and used without contact. In addition to being a safer system, a smaller amount of NaOH and water is needed compared to other systems, there is no need to preheat the water, and the system can even generate heat. As the feeding is drop by drop, the hydrogen flow can be easily controlled by manual or automatic dosing. The waste obtained is solid and contains mostly aluminum hydroxide with some NaOH and impurities from the waste of origin, which are easy to sell and recycle. A study has been carried out to optimize the type of test tubes and establish critical parameters. The results show that a constant and controllable flow rate of hydrogen can be obtained depending on the drip frequency where the chemical reaction predominates over diffusion, that the optimal amount of NaOH is 20 wt%, that a finer grain size can increase the H_2_ yield with respect to the stoichiometric value but reduces the instantaneous flow with respect to that obtained with larger grains, and that it is very important to control the density and the impurities to increase porosity and therefore water diffusion. The estimated cost of the hydrogen produced is 3.15 EUR/kgH_2_ and an energy density of 1.12 kWh/kg was achieved with a test tube of 92% aluminum purity and 20 wt% NaOH.

## 1. Introduction

Currently, there is a lot of interest in the production of hydrogen since it is an energy vector that can be stored more easily than electrical energy, thus increasing the autonomy of mobile devices [1]. Some small-scale mobile devices are Portable Electronic Devices (PEDs). PEDs, due to an increase in power requirements and duration of use, need to guarantee an uninterrupted flow of electrical energy, especially in emergency equipment and in the military sector. Due to long recharge times, battery technology is not viable [2]. Hydrogen technology and fuel cells are future alternatives in equipment where an electrical connection is not available and where high energy efficiency is desired [3]. Direct Carbon Fuel Cells (DCFCs) [4] are fueled by methanol. Some studies indicate that they are the most appropriate method to generate electricity for domestic PEDs due to the difficulty of transporting hydrogen gas. However, due to the slow kinetic rate of the oxygen reduction reaction at the cathode, the need for a fuel pump, and the generation of CO_2_ [5], this fuel cell becomes polluting and expensive, which makes it economically unviable. Polymer Electrolyte Membrane (PEMFC) cells are preferred due to their higher energy density, functionality at room temperature, quick start-up process, compactness, lightness, and low corrosion [6]. These fuel cells are powered by very pure hydrogen to avoid contamination of the platinum catalyst. First of all, the different ways of producing hydrogen will be detailed.

Large facilities are usually used in sustained hydrogen production [7]. There are various types [8]. The most widely used is SMR, due to its lower cost of 3.76–4.41 EUR/kg H_2_, which employ fossil fuel gas reforming, with CO_2_ capture and storage (CCS), to produce hydrogen. In a second type, electricity from renewable energy plants, nuclear or thermal power plants with CO_2_ capture and storage is converted into hydrogen using a PEM, alkaline, or AEM electrolyzer [9]. Finally, there are recent advances in other techniques such as photocatalysis [10] or thermochemical cycles [11] in which light or heat is used to generate hydrogen directly with the help of a chemical agent. The problem with these systems is the impossibility of generating hydrogen in situ, so the hydrogen needs to be stored or depends on the weather.

To power PEMFCs in portable systems, the hydrogen used is transported in metal hydride cartridges or pressurized gas bottles [12]. To adapt this hydrogen transport system to any type of user with PEDs, transport with high-pressure bottles must be ruled out because hydrogen has a high flammability [13], which is why transport with hydrogen bottles is a system considered risky. In the case of metal hydride cartridges, hydrogen is stored in a pressure range of 5–12 bar compared to the 340–700 bar of pressurized bottles, therefore, with greater safety. These hydrogen carrier bottles need to be charged for several minutes at pressures of 10 to 25 bar with pressure bottles or hydrogen generators. Currently, at a commercial level, the system is very expensive and the volumetric density is low under normal conditions. On the other hand, in operation, they need to absorb heat to maintain the hydrogen flow and although research is being carried out to work at lower temperatures (15 °C), the effectiveness decreases and the desorption time, that is, the emission time of a fluid previously adsorbed by a material (desorbed time), increases at lower temperatures [14]. Although one of the main problems with using hydrogen in PED systems continues to be the high cost of fuel cells, due to the use of platinum as a catalyst, manufacturing with economies of scale would reduce the costs of the other elements and make these cells more attractive to users [15]. To improve their use, it is also necessary to develop hydrogen storage or generation systems that are cheap, safe, easy to use by the user, and environmentally sustainable. There are studies to take advantage of recycled aluminum to generate hydrogen through hydrolysis. In some cases, such as from manufacturing processes, it is economically viable, as has been demonstrated [16]. The advantage of hydrogen obtained by the aluminum–water reaction is that hydrogen gas can be produced in situ, at low pressure, and that the byproducts can be recycled with renewable energy to obtain aluminum again, making the system completely sustainable [17]. In aluminum, due to surface oxidation, a passive oxide film is generated that prevents water from instantly reacting with the metal. Upon contact with water, a hydration process occurs in this layer, transforming it all into hydroxide in one of the following chemical forms.

Al_2_O_3_ + H_2_O → 2AlOOH (1)

Al_2_O_3_ + 3H_2_O → 2Al(OH)_3_ (2)

Due to the instability of the hydroxides, a condensation reaction occurs, and aluminum oxide and hydrogen pockets are formed on the internal surface of the aluminum, which subsequently breaks the hydroxide layer that surrounds them, escaping to the outside.

3AlOOH + Al → 2Al_2_O_3_ + 3/2H_2_ ↑ (3)

Al(OH)_3_ + Al → Al_2_O_3_ + 3/2H_2_ ↑ (4)

After this period of time, called the induction time, hydrogen is continuously released as the outside water reacts through the broken hydroxide zone with the aluminum directly. At temperatures below 280 °C, bayerite Al(OH)_3_ is more stable [18].

Al + 2H_2_O → AlOOH + 3/2H_2_ ↑ (5)

Al + 3H_2_O → Al(OH)_3_ + 3/2H_2_ ↑ (6)

In the initial stage, controlled by a surface chemical reaction, the rate of hydrogen production is faster than in the subsequent stages where the byproduct layer thickens and is controlled by the much slower diffusion of water molecules in said layer. The induction time is very long, so attempts have been made to shorten it using different strategies [19,20,21]. *Adding promoters*: this is based on the addition of alkalis or catalysts in the solution [22]. Within the group of alkalis, there are strong alkalis such as NaOH, KOH, LiOH, and Ca(OH)_2_, and weak alkalis such as Na_2_SnO_3_, NaAlO_2_, Na_2_CO_3,_ and Na_2_SiO_3_. Their function is to make the hydroxide layer soluble, allowing the aluminum-water reaction. In the group of catalysts, we find oxides, hydroxides, and transition metal-based catalysts. Their function is to act as catalysts for the hydration process. Regarding the *activation of aluminum process* [23], there are three types: milling, aluminum alloys, and surface modification. The objective is to vary the structure of the aluminum so that it reacts directly with water. In the first type, we perform milling in an inert atmosphere by reducing the particle and combining it with certain compounds (Al + chloride, Al + hydride, Al + carbon-based material, and Al + oxide (hydroxide)). Aluminum alloys use high energy for the milling process (high-energy milling) to promote corrosion, or are melted at high temperatures to form Al-Bi, Al-Sn alloys (high-temperature melting). Finally, surface modification uses phase transformation amalgams with Pb, Sn, In, Bi, Ga or their compounds [24] or a thermo-chemical process, *destroying the layer* by working with nanoparticles and steam at high temperatures [25]. Comparing the different systems, it seems that alkali as a promoter is the most suitable for the case under consideration due to its high production rate, low cost, simple design, and no need for preheating. Disadvantages of this system are strong corrosiveness, difficulty controlling hydrogen production, and production of alkaline wastewater [26]. Other factors that can cause an increase in flow rate are particle size, reaction temperature, pressure, and type of water [27]. Special care must be taken to ensure that the user does not have to handle powdered aluminum since aspiration is harmful to health [28,29], or any other product that could harm their physical integrity. Reactors with this technology have been researched and types have been found: by flooding aluminum in NaOH solution or catalysts [30,31,32,33,34,35,36]; using aluminum microparticles and high-temperature steam [37]; and aluminum activated by metal alloy [38].

It is not believed that they can meet all the requirements that we want for our system, which are user safety, portability, ease of use, purity, low cost, and sustainability.

In the articles consulted, there is no method similar to the one presented here. There are many models that describe how the reaction acts on grains or continuous surfaces but not in models with pressed powder and its parameters, especially with aluminum combined with NaOH. On the other hand, another idea for a research and application route would be the anode-electrolyte of an aluminum–air fuel cell [39].

The objective of the present research is fundamentally to develop a drop-by-drop hydrogen generation system from aluminum specimens in a reactor. To predict the results, a theoretical model had to be constructed for powder specimens combined with NaOH by dripping water.

## 2. Experimental Section

### 2.1. Aluminum, NaOH, and Other Products Used

The specimens have been manufactured by pressing metal powder with recycled aluminum, A1 or A2 type, and NaOH. Table 1 defines the specimens, types A1 and A2. The first type A1 is obtained by mechanical grinding (ball milling) and its size is between 0.1 and 0.5 mm. The second type A2 was obtained by sawing a 2024 aluminum bar and selecting the grains using a 0.7 mm sieve. The shape of the test tubes is cylindrical and the amount of recycled aluminum contained is approximately 20 g. In the description of the experiments, size measurements and weight are specified. NaOH of 98% purity was used.

During manufacturing and storage of the specimens, humidity should be avoided as much as possible, since the mixture reacts with water. When manufacturing the test tubes, work was done with latex gloves and a mask to avoid inhaling aluminum powder and NaOH, as well as the transfer of moisture from breathing. After manufacturing the test tubes, they must be transported in an airtight container. In the tests, tap water was used. Salt water should be avoided as it could clog the pipes. The desiccant used to dry the hydrogen before measuring its flow rate in the flowmeter was drying pearls of silica gel with a moisture blue indicator.

### 2.2. Devices for Manufacturing Test Specimens and Measuring Hydrogen Flow

**Figure 1:**
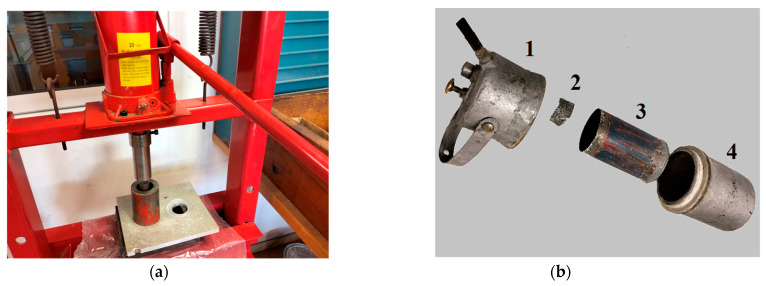
Press and die to press the specimen of Al + NaOH to obtain test tube (**a**); disassembled carburetor (**b**), showing the upper water tank 1, Al + NaOH test tube 2, sheet steel container 3, and reactor 4.

In the manufacture of the specimens, a 20 Tn manual press was used (Figure 1a). To carry out the measurements of the dimensions and weight of the test pieces, a 150 mm × 0.01 mm digital Vernier caliper, and a 500 g/0.01 g precision scale were used. To measure the instantaneous and total flow rate of hydrogen, a Mass Flow Meter GFM17 of flow range 0–10 L/min with an AALBORG Totalizer was used.

### 2.3. Method of Obtaining Hydrogen

**Figure 2:**
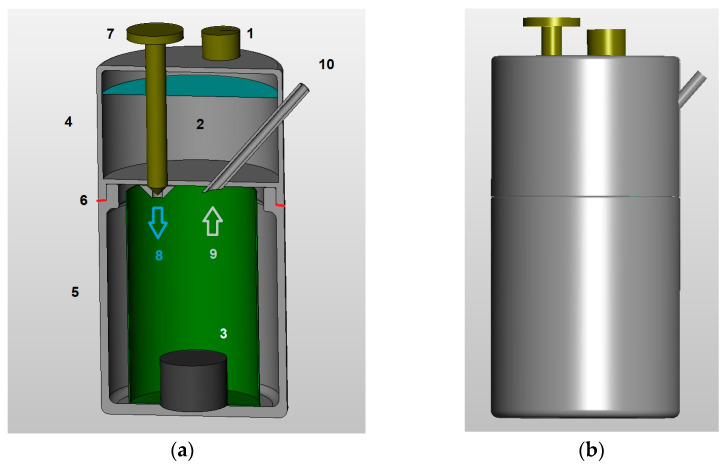
Scale drawing of the carburetor used in the tests. Apparatus in section (**a**): tank water cap 1, water 2, Al + NaOH test tube 3, upper tank 4, reactor 5, gasket 6, dosing screw 7, water drops input 8, output of generated hydrogen 9, hydrogen output metal tube 10. Exterior view (**b**).

To obtain hydrogen, a drop-by-drop system [40] was used. Figure 1b shows the main elements of the apparatus. The carburetor consists of two main parts, an upper water tank (1) and a reactor (4), that are joined by threading one piece onto the other after inserting a sheet steel container (3), with Al + NaOH test tube (2) inside, in the reactor (4). Once assembled, as shown in Figure 2b, the device measured 160 mm high and 75 mm in diameter. A sheet steel container (3), which measured 95 mm high by 53 mm in diameter, was added to facilitate the introduction of the Al + NaOH test tube (2), protect the reactor (4), and also facilitate the emptying of waste once the reaction has finished. Optionally, an airtight lid could also be added to said container (3) to transport the Al + NaOH test tube to the laboratory from the factory. The user could buy the sheet metal container and after that remove the lid and insert it with the specimen in the reactor (4). In the same way, after completing the reaction, the container was removed, after closing the lid, to transport the waste to the waste manager.

#### 2.3.1. Description and Initial Preparation

Figure 2a describes the apparatus in the section. A sheet steel container, where Al + NaOH test tube (3) has been deposited, is introduced into the reactor (5). This reactor (5) is then screwed to the upper tank (4), introducing a Teflon gasket (6) to ensure the tightness of the connection. The water is introduced into the tank (4) through the opening where the cap (1) is. A screw (7) controls and doses the water drops (8). Initially, this screw is completely tight so it prevents the water from escaping.

#### 2.3.2. Working Mode

**Figure 3:**
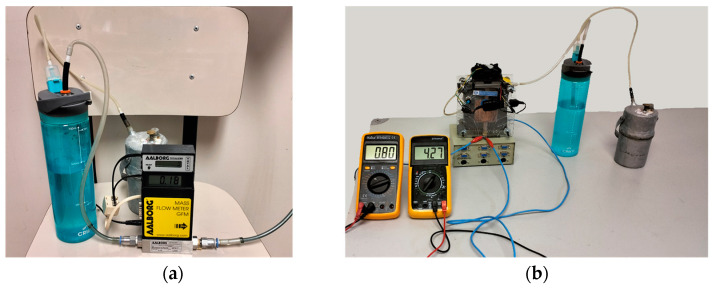
Assembly for H_2_ flow measurement showing the connection between the carburetor, the bubbler, and the flowmeter (**a**). Direct connection assembly with a Fuel Cell charging a mobile phone battery (3.7 V 4500 mAh Li-ion Li Po) at 4.2 V, showing the intensity 0.8 A and voltage 4.27 V on the multimeters (**b**).

As shown in Figure 2a, when adjusting the screw (7), the water (2) located in the upper tank (4) is introduced drop by drop (8) into a sheet steel container in the reactor (5). When the water comes into contact with the Al + NaOH test tube (3), hydrogen is produced (9), which due to its lower density moves through the metal tube (10) to the outside. Afterward, as shown in Figure 3a, it is directed through a flexible tube attached to a bubbler where the hydrogen is cleaned and after passing through a gas dryer with silica gel it is taken to the flow meter, storage tank, or fuel cell (Figure 3b).

Depending on the regulation of the screw, the drip frequency and therefore the hydrogen flow will vary. It must be taken into account that at the beginning, the reactor is at room temperature, and since the speed of the reaction depends on the temperature, the reaction will initially be very slow; not all the water added by dripping will be consumed, and the excess will accumulate in the reactor. When the reactor is heated to a critical temperature close to 60 °C, the reaction speed will increase exponentially and all the accumulated water will immediately be transformed into hydrogen, which will drag along the aluminum hydroxide and the aluminum dust released, even clogging the tube exit. To avoid this, a small amount of water must be initially poured until the reactor heats up, thus avoiding overflow of the system. During the process, the water must be dosed correctly as it could pool and react abruptly with the increase in temperature. This will establish the maximum hydrogen flow rate depending on the size and type of specimen. The reaction rate r (Equation (8)) between Al and H_2_O depends on the rate constant k and the orders of reaction, m and n, obtained experimentally. The rate constant k (Equation (7)) has an exponential relationship with the temperature T (Equation (7)), where *Ea* is the activation energy and R is the universal gas constant [41].

(7) $$k\left(T\right)=Ae^{-\frac{Ea}{RT}}$$

(8) $${r=k\left[Al\right]}^{m}{\left[H_{2}O\right]}^{n}$$

#### 2.3.3. Previous Tests

Some preliminary tests were carried out to study how the system behaves inside the carburetor. The upper tank was filled with water and an Al + NaOH test tube with chemical composition aluminum type A1 with NaOH, 16.67 wt% was placed inside. Subsequently, the screw was regulated in such a way that throughout the test, the dripping frequency was nine drops per minute. Each drop has a volume of 0.065 mL, so the drip flow rate was 0.585 mL/min. This constant flow rate with a high interval between drips was chosen to try to characterize the model of the reaction of a drop on the surface. During the test, the water level in the upper tank was kept constant to ensure drip frequency. The test was completed after 29 min to evaluate the behavior of the system in that period by measuring the instantaneous and total hydrogen flow rate and the temperature of the water in the upper tank. Table 2 describes the Al + NaOH test tube used and the flow rate supplied.

**Figure 4:**
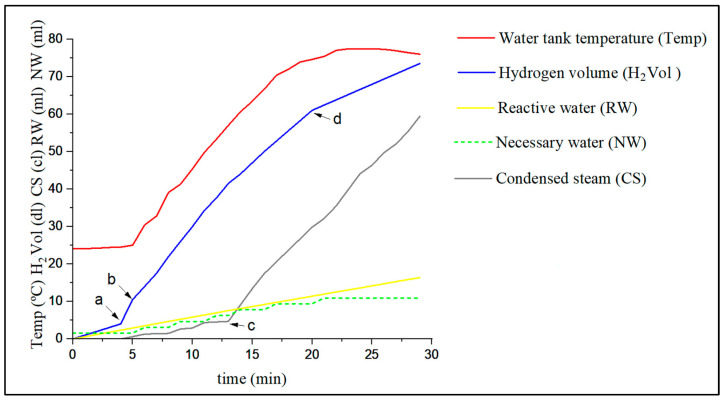
Parameters of the preliminary test.

At the end of the test, the weights of the bubbler and the lower tank were compared with the initial ones to control the vapor that condenses in the system and therefore does not react. Figure 4 shows the water needed in ml to produce 1 additional liter of hydrogen (NW), and the water that reacts in ml with the test tube (RW) as well as the steam generated in cl (CS). On the other hand, the hydrogen generated in l (H_2_ Vol) and the temperature of the upper tank water (Temp) described in Figure 2a, is represented.

After 4 min (Figure 4a), a temperature is reached at which diffusion is complete for the aluminum-water reaction to occur, and in a few seconds the hydrogen flow increases drastically since the water that can react accumulates (RW) and exceeds the water needed to obtain an additional liter (NW). At that moment, the accumulated water disappears and the flow of hydrogen (H_2_ Vol) stabilizes at minute 5 (Figure 4b), because the flow of water introduced is transformed almost entirely into hydrogen (H_2_ Vol). Slowly the temperature of the reactor, test tube, and water in the upper tank (Temp) increases and water vapor (CS) begins to form, reducing the amount of water that reacts with the aluminum surface (RW). After 13 min (Figure 4c), the temperature of the water in the tank and reactor is so high that a large part of the water evaporates (CS) and the reactant water (RW) is less than the necessary water (NW). Therefore, the flow rate slows down after 20 min, as does the generation of hydrogen (Figure 4d). It can be seen that the temperature of the water in the tank, lower than that of the reactor, reaches 78 °C. Given the results, we could define a theoretical model between the period between 5 and 13 min, which is where the accumulated water and the steam generated are insignificant. In this section, the flow of hydrogen generated is a product of the water introduced by dripping. It is observed that in the section between 13 and 20 min, although the amount of steam generated is greater due to the reactor temperature, the hydrogen flow rate remains constant due to the accumulated water. Therefore, one way to keep the hydrogen flow rate constant, without complicating the design, is to introduce more water than necessary to avoid total evaporation on the surface of the test tube, while avoiding excess, since there can be a lot of water. Accumulated water and overheating, due to the reactions of equations (Equations (9) and (10)), can lead to a sudden excess of generated hydrogen which drags the aluminum powder and aluminum hydroxide to the outside of the tube.

NaOH (s) → Na^+^ (aq) + OH^−^ (aq)    ∆H = −44.51 kJ/mol (9)

Al + 3H_2_O → Al(OH)_3_ + 3/2 H_2_ ↑   ∆H = −415.5 kJ/mol Al (10)

It can be observed that in the interval between 5 and 13 min in the graph in Figure 4, the line maintains an approximately constant slope. This means [42] that we are in an area where the chemical reaction predominates over diffusion since this, due to the temperature of the reaction, is much faster since, according to the Arrhenius equation, the kinetics grow exponentially [43]. It follows a stoichiometric law and the hydrogen generated corresponds to the water supplied.

#### 2.3.4. Theoretical Model

In the present investigation, a theoretical model has been defined to analyze the behavior of powder specimens pressed with NaOH under dripping water. As a hypothesis, the macroscopic model considers a low drip frequency. The process studied describes the initial and final phase of the proposed model.

### 2.4. Initial Phase

**Figure 5:**
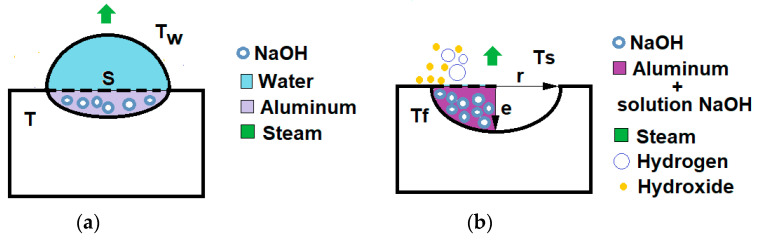
Evolution of the aluminum–water reaction in the test tube formed of active aluminum with NaOH and a drop of water. Initial phase (**a**), final phase (**b**).

In this phase, shown in Figure 5a, the drop falls on the sample of pressed aluminum powder activated with NaOH. In order that water diffusion does not occur only on the surface of the specimen, where there is little NaOH, the surface S and the rest of the specimen must have a certain porosity. As the water diffuses through the pores of the test tube, the solid NaOH particles are solubilized according to Equation (7), generating an increase in enthalpy ∆H and heating the solution. Ti is the initial temperature of the drop, and its final temperature can be found using the formula (Equation (11)).

(11) $$Tf=\frac{∆H*\;n_{NaOH\;\;}}{Cp*\;m_{tot}}+Ti$$

where *n_NaOH_* is the number of moles of NaOH that intervene locally, ∆*H* is the increase in enthalpy −44.51 kJ/mol, *m_tot_* is the total mass in the solution which is the sum of the mass of NaOH and H_2_O, and *Cp* is the heat capacity of H_2_O, 4.18 kJ/kg °C. Initially, the initial temperature Ti = Tw which is the temperature of the water drop, but due to the solubility reaction and the aluminum–water reaction, the specimen is heated to the temperature T and transfers part of the heat to the water drop, a fact that modifies its Ti and can even transform part of it into steam. An advantage of this system is that, although the amount of NaOH distributed homogeneously in the test tube is small, as the amount of water is also small, the molarity of the solution and therefore the pH is high.

### 2.5. Final Phase

In this phase, NaOH solution at temperature Tf reacts with the aluminum, hydrating the passive film layers of the grains (Equation (2)), and eliminating the solid aluminum hydroxide layers by dissolving them as shown in Equation (12), to form NaAl(OH)_4_.

Al(OH)_3_ (s) + NaOH → Na Al(OH)_4_ (aq) (12)

Once the passive layers have been eliminated, the water reacts with the aluminum generating hydrogen and aluminum hydroxide by the aluminum–water reaction according to Equation (10) and heat, due to the increase in enthalpy. Said increase in enthalpy varies between 415.5 kJ/mol Al at 0 °C up to 426 kJ/mol Al at 100 °C; the average value will be taken as 420.75 kJ/mol Al. This heat will be transmitted to the test tube, to the hydrogen generated, to possible accumulated water, and to the reactor by conduction. In the case of the specific reaction zone, the temperature reached Tf2 from the initial temperature of this zone Tip, after an initial phase, can be calculated with Equation (13).

(13) $${Tf}_{2}=\frac{∆H*\;n_{Al\;\;}}{c*\;m_{tot}}+Tip$$

where *n_Al_* is the number of moles of Al that intervene locally, ∆H is the increase in enthalpy 420.75 kJ/mol_Al_, *m_tot_* is the total mass included in the reaction volume delimited by the zone that is wetted by water and, given that the mass total is composed of more than 75% aluminum, the chosen specific heat c is that of aluminum, i.e., 0.9 kJ/kg °C.

As hydrogen is formed, more aluminum hydroxide will be produced and since the amount of NaOH is reduced, precipitating aluminum hydroxide will be generated as indicated in Equation (14). Said precipitate will be transported by the hydrogen bubbles themselves away from the reaction zone. Sometimes this same hydrogen will decompose the test tube.

Na Al(OH)_4_ (aq) → Al(OH)_3_ (s) + NaOH (14)

We will assume that the volume wetted by the drop corresponds to the spherical cap in Figure 5b, the average surface of which, measured experimentally on a specimen with the carburetor, is 95 mm^2^. So, the radius of the circular surface is r = 5.5 mm. The volume as a function of r and the height of the cap e is indicated in Equation (15) and does not have to be equal to the volume of the drop since the liquid penetrates through the pores:

(15) $$V=\frac{\pi *e}{6}\;\left(3*r^{2}+e^{2}\right)$$

**Figure 6:**
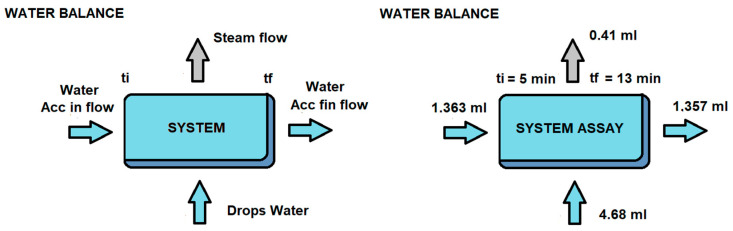
Balance of water that reacts with the test tube at the chosen interval between 5 and 13 min.

To calculate the height of the cap, the amount of material that has reacted must be calculated. Figure 6 shows the balance of the water that exists in the system in the chosen time interval between 5 and 13 min of Figure 4.

The incoming water comprises the accumulated water that has not reacted in the first 5 min plus the water supplied from the upper tank, while the outgoing water is the water accumulated in the 13th minute that has not reacted plus the water vapor that does not return to the system. The total amount of water that will be generated by the hydrogen produced in the interval is 4.645 mL.

Once the volume of water that enters the system has been obtained, we will calculate by stoichiometry (Equation (16)) according to Equation (10) the hydrogen obtained, taking into account that it has been measured in the flowmeter at 25 °C and 1 atm, so its density is approximately 0.0824 g/mL H_2_.

(16) $$4.645\;\mathrm{mL}\;H_{2}O+\frac{1\;g\;H_{2}O\;}{1\;mL\;H_{2}O}+\frac{1\;mol\;H_{2}O}{18\;g\;H_{2}O}+\frac{1.5\;mol\;H_{2}}{3\;mol\;H_{2}O}+\frac{2\;g\;H_{2}}{1\;mol\;H_{2}}+\frac{1\;mL\;H_{2}}{0.0824\;g\;H_{2}}=3.13\;mL\;H_{2}\;\;\;\;\;$$

In the flowmeter, for this interval, a real flow rate of 3.1 mL H_2_ was measured, so the value given by stoichiometry is similar to that obtained experimentally. As can be seen from Figure 6, the water flow rate in the system is approximately supplied in that interval by the carburetor, so the H_2_ flow rate can be controlled by water dosing. As the water flow rate has been carried out by supplying nine drops for 8 min, the average flow rate of each drop deposited on the surface is 0.0645 g of H_2_O per drop deposited. Stoichiometry can be used in the same way to calculate the amount of material that is in the wet volume of Figure 5b, knowing that aluminum is 97% pure, NaOH is 98% pure, and that the amount of NaOH is 16.67 wt% of the test tube. From the density of the material (Table 2), the wetted volume can be calculated and, using Equation (13), the value of the cap height e can be calculated. On the other hand, from the volume of water and the amount of pure NaOH, the volume of the solution and the molarity of the NaOH solution in the drop could be obtained.

It can be highlighted in Table 3 that the molarity of the drop when produced locally is very high in comparison with NaOH introduced into the test tube, which together with the temperature generated by dissolution will produce a diffusion of the liquid and a rapid reaction. To achieve the same effect in 100 mL of water by immersion, you would need about 10 g of NaOH instead of 4 g and preheating.

### 2.6. Study Parameters

Next, the study parameters that can influence the production of hydrogen in the proposed generator are defined. The first would be the flow rate of added water or drip frequency and the second would be the type of aluminum, purity, and alloy elements of the source material. To focus the study in this research, only one type of aluminum alloy was studied as the active aluminum specimen. The parameters to analyze are the following:

- Drip frequency.
- Percentage of NaOH and the amount of aluminum in the test tube.
- Aluminum grain size.
- Density and porosity of the test piece.

## 3. Assays and Methodology Used in Tests

### General Methodology

In all tests, the carburetor described will be used with its upper tank initially filled with tap water, with a level height of h = 50 mm. Subsequently, water is added drop by drop to the test tube, filling the upper tank each time a liter of hydrogen has been generated to ensure that the dripping frequency does not vary due to the variation in water height [44].

The hydrogen generated is introduced into a bubbler with distilled water for cleaning, which will have a constant level height of 4–6 mm. In the event of excess steam condensation in the bubbler that increases said height, the excess will be extracted through a valve. The clean hydrogen, after drying, is introduced into a flowmeter with a totalizer to measure its flow. In general, except when describing the grain size effect, type A2 aluminum was used.

The study includes the time taken to generate each additional liter of hydrogen. Special care must be taken when interpreting the data at the beginning of the reaction, since, as mentioned, due to the transition in the reaction zone of the specimen from the initial ambient temperature to the higher working temperature, the accumulated water flow reacts abruptly, reaching in some cases instantaneous flow rates of up to 2 L per minute for a few seconds. Once the working temperature is reached, the flow rate becomes constant.

## 4. Results and Discussion

### 4.1. Results with Different Drip Frequencies

**Figure 7:**
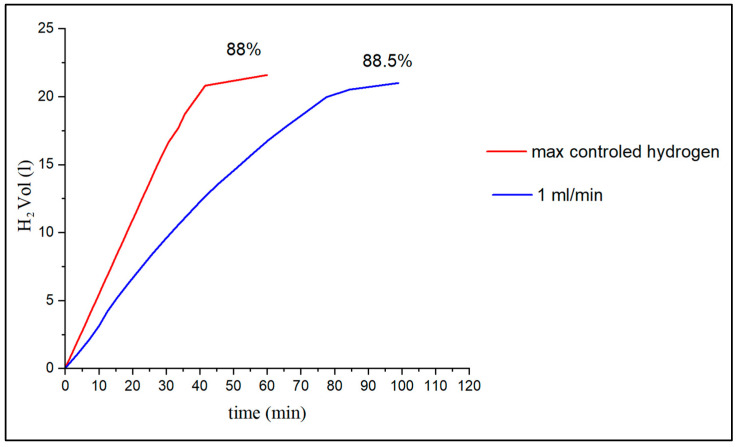
Effect of the dosed flow rate of water in A2 aluminum specimens with 20 wt% NaOH.

When working with flow rates in which the necessary volume of water is guaranteed, but where an excess of hydrogen does not occur due to water accumulation, the flow of hydrogen generated is proportional to the dosed flow. Figure 7 shows the flow rate produced in a test tube of approximately 24 g of type A2 aluminum mixed with 20 wt% NaOH for different drip rates. In the case of max-controlled hydrogen, the drip flow rate has been regulated between different flow rates, 1–1.2–1.5 mL/min, depending on the instantaneous hydrogen flow rate to achieve the maximum hydrogen flow rate in a controlled manner. In the second case, a test tube similar to the previous one was chosen, but it was dosed at a constant drip flow rate of 1 mL/min. As can be seen, depending on the drip rate of water provided, a variable hydrogen flow rate can be achieved. From a certain point on, the aluminum surface is too small and diffusion predominates over the chemical reaction on the surface on the grains, and the flow rate of hydrogen generated is very low. Finally, the H_2_ yield for this type of test tube is usually 88–89% in these conditions compared to the stoichiometric value. From the results, we deduce that constant flow rates can be generated in a controlled manner by varying the drip frequency.

### 4.2. Percentage of NaOH and the Amount of Aluminum in the Test Tube

Different types of A2 aluminum specimens were manufactured by varying the percentage of NaOH, and these were tested with the carburetor to obtain the maximum controlled hydrogen flow. Table 4 shows a summary of their behavior.

**Figure 8:**
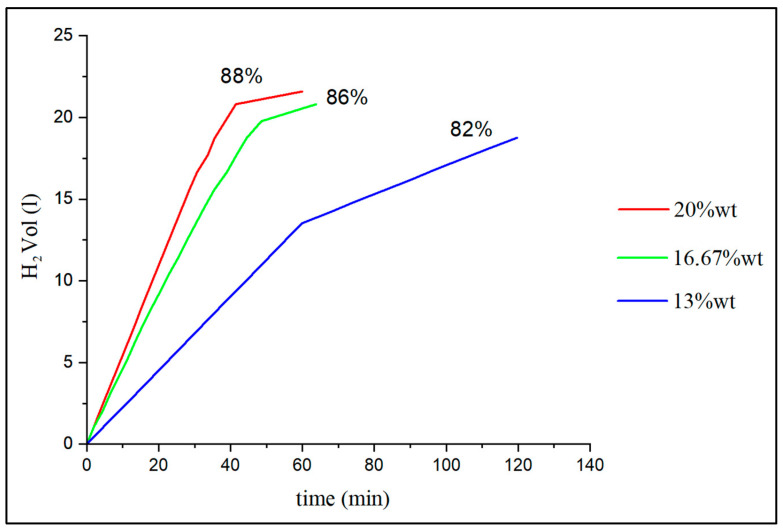
Effect of the proportion of NaOH in the Al + NaOH test tube.

It can be observed that the test tubes work correctly for percentages of 13 wt%, 16.67 wt%, and 20 wt%. In the case of 9 wt%, it is found that there is not enough NaOH to start the system and the test tube ends up flooding. In the cases of 23 and 28 wt%, the problem is the loss of porosity of the specimen as the reaction progresses [31]. As will be seen later, for the system to work, the liquid must reach the appropriate volume and be wet with water. Between the three percentages of functional NaOH, a comparison is made of the kinetics of the controlled reaction and the hydrogen yield, that is, the percentage of hydrogen obtained relative to the stoichiometric value depending on the amount of pure aluminum in each test tube, in Figure 8. The characteristics of the aluminum specimens tested and the amount of hydrogen obtained are shown in Table 5.

Figure 8 shows that the highest flow rate and the best hydrogen yield are obtained with 20 wt% of NaOH, but the difference with 16.67 wt% is not too significant, so if the objective were to reduce the amount of NaOH the second option would be preferred. For 13 wt%, the hydrogen yield drops significantly to 82% and a smaller slope is observed. Logically, the more test tubes there are in the reactor, the more active aluminum surface there will be, and therefore more accumulated water will be converted into hydrogen, which can increase the frequency of dripping without overflow and increase the slope of the graph curves, although not the hydrogen yield. The result is that the best option is NaOH 20 wt% of a test tube with Al aluminum of Table 1.

The instantaneous flow in the area of maximum slope is shown in Table 6.

### 4.3. Practical Application

In a low-cost commercial FC such as a Horizon 20 FC, the required instantaneous flow rate is 0.014 L/minW [45]. A very common range of batteries is studied, 4500 Ah at 3.7 V. These batteries can be charged slowly or with fast charging. The case that requires greater power is fast charging in which, according to the manufacturers, 60% of the battery is charged in 30 min, and for this 22.5 Wh chargers are used. For an FC Horizon 20, the power of 22.5 Wh can be achieved with a hydrogen flow rate of 0.315 mL/min, so it would be sufficient to use the 16.67 or 20 wt% test tubes. The total volume of hydrogen generated of 21 L would be enough to fully charge the battery. Figure 3b shows the charging of a battery of this type using the proposed hydrogen generation system and a self-built 20 W fuel cell. Extrapolating the data, the energy density would be 1.12 kWh/kg of test tube type A2, with 20 wt% test tube, without counting the weight of the carburetor, bubbler, and fuel cell.

### 4.4. Aluminum Grain Size

Generally, by decreasing the aluminum grain size, the hydrogen yield obtained increases [46]. However, in this case, the grains are compacted and the reaction increases with the volume wetted. If density increases, porosity decreases more in smaller grains than in larger ones and wetted volume is minor.

**Figure 9:**
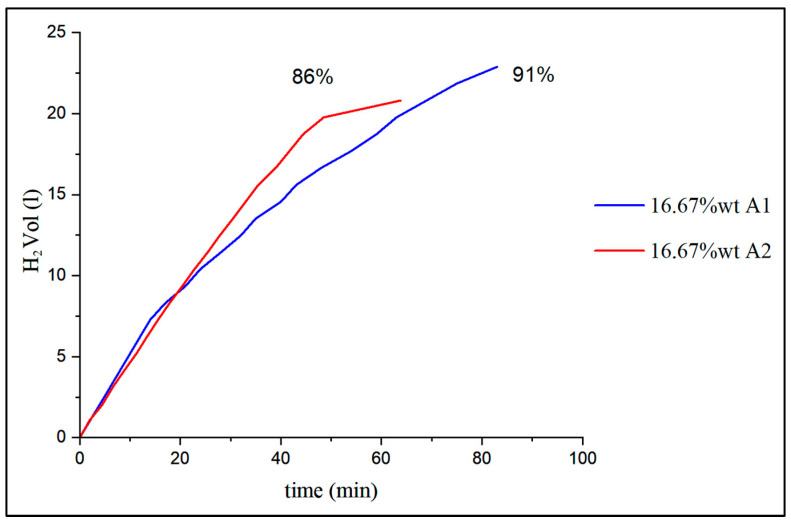
Flow rate obtained for specimens with the same proportion of 16.67 wt% NaOH for different types of recycled aluminum with smaller grain size A1 and larger size A2.

Figure 9 shows that, initially, the behavior is as expected and the instantaneous flow rate is greater for smaller grains that also have a higher percentage of pure aluminum. After 20 min, the instantaneous flow rate is greater in the larger grain size specimens and from the study of the condensed water, it does not seem to be due to the steam generated. It was only possible to deduce, from the tests with specimens with a higher and lower percentage of NaOH, as seen in Table 7, that in all cases the hydrogen yield is higher and that the same phenomenon occurs. For future studies, we should work with aluminum of the same type and different grain sizes; such means were not available at the time of the tests. One hypothesis, as will be seen in the next section, is that specimens with smaller grains are more likely to form clumps of material as density increases, so decreasing porosity slows down diffusion. On the other hand, a grain with a smaller diameter will decompose faster than one with a larger diameter, and therefore the generation of total hydrogen and thus yield will be greater.

The result is that the instantaneous flow rate is greater in pressed Al + NaOH with larger grains but the hydrogen yield is higher with smaller grains.

### 4.5. Density and Porosity in the Specimen

**Figure 10:**
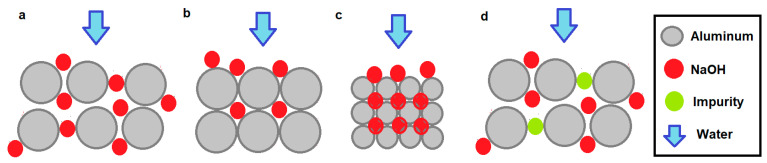
(**a**) Test tube with low density and high porosity. (**b**) Specimen with medium density and medium porosity. (**c**) Specimen with high density and low porosity. (**d**) Test tube with low density and low porosity.

In general, specimens with low density, that is, with spaces between the grains due to less compaction, allow water and NaOH solutions to pass through better, and therefore the diffusion, reactivity, and generation of hydrogen are greater, as can be seen in Figure 10a, where water will easily dissolve the NaOH grains. But we must also take into account porosity [46]. In Figure 10d, the density is low, but some impurities prevent the passage of water or solution and therefore inhibit diffusion. In some cases, this is caused by the NaOH itself, which if it is in high concentrations forms a paste before dissolving that prevents diffusion. In these cases, the solution must react and reduce the walls of aluminum grains to be able to pass through, which slows down the generation of hydrogen. If the density is high, that is, the grains are very close together due to greater compaction as shown in Figure 10b, the smaller space between them makes the porosity medium and diffusion difficult. In these cases, the grains must be reduced to allow the solution to pass, but if there are larger grains there are fewer partitions and therefore the diffusion will be greater than with small grains. In this same case, if the amount of NaOH is greater, but without changing the porosity, it will reduce the grains sooner and the diffusion will be faster. In the case in which the density is greater but the grains are smaller, Figure 10c, there will be more walls to reduce to reach the same surface area and therefore the diffusion will be less. If the amount of NaOH is greater, but without changing the porosity, the diffusion will be faster. As mentioned in the previous section, when the solution has reached all the grains, the small grains dissolve better than the large ones (Figure 10b,c) and end up generating a greater total flow of hydrogen, that is, the hydrogen yield is greater.

When working with a higher concentration of NaOH, as long as it does not affect the porosity, you can work with higher densities without affecting the infusibility. This allows working with smaller volume specimens containing the same amount of aluminum to be reacted, but their weight increases due to the greater amount of NaOH. Table 8 shows the density values that have worked for the different types of specimens or percentages of NaOH. To achieve a certain density, the press height must be controlled. Too low a density could mean that the specimen may disintegrate with use and may be more affected by humidity.

The effect of introducing an additional catalytic promoter to NaOH, such as an oxide or a salt, has also been tested. It is not recommended to introduce the promoter inside the test tube since introducing an impurity would reduce porosity; even in the best case it does not add any improvement. The way to introduce the catalyst is to dissolve it in the water in the upper tank. In this case, an Fe_2_SO_4_ salt (0.6 g/L) was used which allows working with higher specimen densities for this range. As we do not want to introduce any additive to the tank water to guarantee the safety of the user, this is indicated only for future studies.

Generally, the results indicate that the lower the porosity of the pressed Al + NaOH specimens, the lower the instantaneous flow rate. This porosity can decrease by increasing the density or by adding impurities that are not easily soluble. Density may be higher in specimens with a higher percentage of NaOH, maintaining an optimal instantaneous flow, within the range marked in Section 4.2.

### 4.6. Purity and Cost Analysis

The purity of the hydrogen obtained [9] with the aluminum–water system, once purged of air, is greater than 99%. It does not contain undesirable gas or alkalis but its humidity is high (>1%), especially at high flow rates, which requires drying systems, as moisture can affect the performance of the fuel cell. The cost analysis will be carried out for type A2 aluminum with 20 wt% NaOH.

2Al(OH)_3_ − 3H_2_O → Al_2_O_3_ (17)

**Figure 11:**
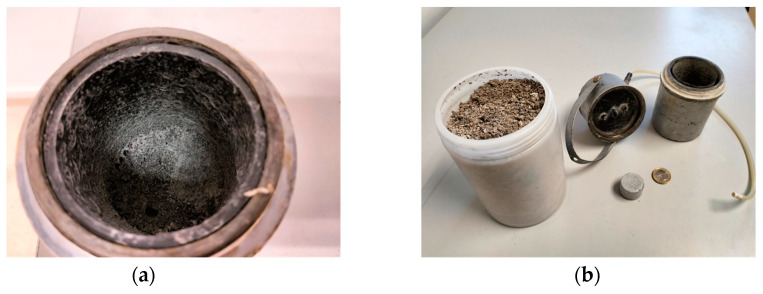
By-product obtained in the reactor (**a**). Stored residues from various tests (**b**).

The cost is low if it is obtained on a large scale since the test pieces are made of recycled aluminum, the NaOH is recycled and the by-product (Figure 11) can be sold to be transformed into high-quality first-cast aluminum, using the Bayer [47] method (Equation (17)). The economic study includes the price of aluminum scrap from turning (London Scrap Metal Price Nov 2024) of 0.26–0.36 EUR/kg, and the price of Al_2_O_3_ (COMEX Alumina Nov 2024) of 0.4–0.5 EUR/kg Al_2_O_3_, but due to the distributor’s management and transportation costs, the sales price of turning aluminum scrap is 0.6 EUR/kg and a purchase price of Al_2_O_3_ is 0.3 EUR/kg. The cost of preparation of aluminum specimens is 0.53 EUR/kg H_2;_ the cost of waste preparation by the Bayer method [48] is 1.5 EUR/kg H_2_ and the management cost is 0.25 EUR/kg H_2_ [49]. The estimated cost per kg is shown in Table 9.

## 5. Conclusions

A new method that works with powder-pressed recycled aluminum turnings activated with small amounts of NaOH and drops of water has been described and tested with a carburetor. Assuming that the user buys the capsules or test tubes, they only have to add water, regulate the dosage manually or automatically, and connect it to a fuel cell to be able to charge electronic devices in isolated areas. The system is sustainable since the by-product is solid and can be easily stored, and it can be recycled to obtain first-melt aluminum again or other products that require alumina. A theoretical model has been created to explain its operation and the fundamental parameters. The results show that the dosed water flow rate in these systems must be higher than the stoichiometric flow rate due to the loss due to steam formation, that a constant and controllable flow rate of hydrogen can be obtained depending on the drip frequency where the chemical reaction predominates over diffusion, that the optimal amount of NaOH is 20 wt%, that a finer grain size can increase the H_2_ yield compared to the stoichiometric process but reduces the instantaneous flow with respect to larger grain size, and that it is very important to control the density and the impurities to increase porosity and therefore water diffusion. The estimated cost of hydrogen produced is 3.15 EUR/kgH_2_ and an energy density of 1.12 kWh/kg was achieved with a test tube with 92% aluminum purity and 20 wt% NaOH. With a test tube of about 20 g, the system can generate enough flow to supply hydrogen to a fuel cell and perform rapid charging of a commonly used battery. Depending on the test tubes added, there is more or less autonomy and it is very easy to regulate the desired flow rate to be able to generate the necessary hydrogen depending on the loads required. Finally, results indicate that the system can produce heat so it could be used to heat water in isolated places. Ultimately, this research contributes to developing a completely new system for producing hydrogen that can be used in isolated environments and can contribute to charging electrical appliances and batteries in isolated areas or environments isolated from the electrical grid. Future research will focus on increasing the flow rate and amount of aluminum produced, and looking for applications where water does not have to be transported, as in ships.

## Figures and Tables

**Table 1:** The chemical composition of the aluminum in the test pieces.

Aluminum	Si (%)	Cu (%)	Zn (%)	Fe (%)	Mg (%)	Mn (%)	C (%)	O_2_ (%)	Others (%)	Al (%)
A1	<0.4	<0.4	<0.4	<0.4	<0.4	<0.4	<0.4	<0.3	<0.5	97
A2	<0.5	<4.9	-	-	<1.8	<0.9	-	-	<0.15	92

**Table 2:** Pretest parameters. Type of aluminum and NaOH wt%; characteristics of the cylindrical tested tubes (height, diameter, weight, and density), amount of aluminum and pure NaOH, and water flow (drop by drop).

Type of Aluminum	NaOH wt%	Height (mm)	Diameter (mm)	Weight (g)	Density (g/cm^3^)	Aluminum 100% (g)	NaOH 100% (g)	Flow (mL/min)
A1	16.67	19.2	27.24	23.59	2.108	19.07	3.85	0.585

**Table 3:** Calculated parameters for the wetted volume for a drop of Figure 5b (mass, volume, cap height e), amount of pure aluminum and NaOH, and molarity of the NaOH solution.

Mass of Wet Volume (g)	Volume (mm^3^)	e (mm)	Al 100% (g)	NaOH 100% (g)	M NaOH (mol/L)
0.0399	17.657	0.37	0.03225	0.00651	2.3

**Table 4:** Effect of the percentage of NaOH in the Al + NaOH test tube.

9 wt%	13 wt%	16.67 wt%	20 wt%	23 wt%	28 wt%
Slow	ok	ok	ok	Incomplete	Incomplete

**Table 5:** Characteristics of the tested tubes (height, diameter, weight and density), amount of aluminum and pure NaOH they contain, and hydrogen generated.

NaOH (wt%)	Height (mm)	Diameter (mm)	Weight (g)	Density (g/cm^3^)	Al (100%) (g)	NaOH (100%) (g)	Weight (g)	Hydrogen (ml)
20	20.86	27.29	24.76	2.029	18.22	4.85	24.76	21,600
16.67	22.62	27.02	23.50	1.812	18.02	3.83	23.50	20,800
13	20.30	27.20	21.20	1.797	16.96	2.71	21.20	18,800

**Table 6:** Instantaneous flow rate for the different specimens and comparison of FC in mobile fast charging.

13 wt% (mL/min)	16.67 wt% (mL/min)	20 wt% (mL/min)	FC Flow 22.5 Wh (mL/min)
0.23–0.14	0.45–0.38	0.55–0.35	0.315

**Table 7:** Yield of hydrogen generated for different grain sizes and NaOH wt%.

Sample Composition	13 wt% (%)	16.67 wt% (%)	20 wt% (%)
A1 (smaller grains)	86	91	92
A2 (larger grains)	82	86	88

**Table 8:** Densities of the functional specimens depending on the type of material and percentage of NaOH.

Samples Composition	13 wt% (g/cm^3^)	16.67 wt% (g/cm^3^)	20 wt% (g/cm^3^)
A1	1.79–1.9	1.8–2.12	2.2–2.21
A2	1.79–1.84	1.61–181	1.99–2.03

**Table 9:** Estimated cost per kg of hydrogen in the case of aluminum type A2 with 20 wt% NaOH.

Al (92%) [kg/kg H_2_]	Al_2_O_3_ [kg/kg H_2_]	Cost Preparation and Management EUR/kg H_2_]	Cost Al Scrap [EUR/kg H_2_]	Sale Al_2_O_3_ [EUR/kg H_2_]	Price EUR/kg H_2_
11.12	19.33	2.28	6.67	5.8	3.15

## Data Availability

The original contributions presented in the study are included in the article, further inquiries can be directed to the corresponding authors.
